# Submitral valve aneurysm in Bulawayo, Zimbabwe: A case report

**DOI:** 10.1002/ccr3.2202

**Published:** 2019-05-10

**Authors:** Rumbidzai Chineka, Mark Dixon, Narcisious Dzvanga

**Affiliations:** ^1^ United Bulawayo Hospitals Bulawayo Zimbabwe; ^2^ Faculty of Medicine National University of Science and Technology Bulawayo Zimbabwe

**Keywords:** heart failure, mitral regurgitation, subvalvular aneurysm

## Abstract

Submitral valve aneurysms are a rare but well‐known pathology. These aneurysms should be considered as a differential diagnosis in young individuals who present with symptoms of heart failure and mitral regurgitation.

## 1 INTRODUCTION

Submitral valve aneurysms are a rare cardiac pathology. They are thought to be a congenital abnormality particularly common in black Africans. Submitral valve aneurysms may come about as a result of an inflammatory process such as human immunodeficiency virus (HIV), tuberculosis, infective endocarditis, and rarely Takayasu arteritis.^1^, ^2^ The case reported here is that of a young male patient with a submitral valve aneurysm presenting in congestive cardiac failure.

## 2 CASE REPORT

We are presenting a 24‐year‐old male patient who, 1 and 1/2 years prior to presentation at our institution noticed swelling of his feet, had New York Heart Association (NYHA) three symptoms, paroxysmal nocturnal dyspnea, and a 4‐pillow orthopnea. The patient had a transthoracic echocardiogram (TTE) done 2 months prior to admission at another institution which showed an ejection fraction of 50% and mitral regurgitation and tricuspid regurgitation and was subsequently commenced on frusemide 80 mg orally once daily, spironolactone 25 mg orally once daily, and captopril 25 mg orally three times daily. When he presented to us, he had been having hemoptysis and episodes of fever. The patient had no history of rheumatic fever as a child or any known congenital cardiac condition. He did have a chronic cough as a child which was not investigated. The patient denied smoking and taking alcohol and was not an intravenous drug user. His last positive TB contact had been 5 years prior to admission. The patient was unemployed presently but had worked as an illegal gold miner for 7 years.

On examination, he was ill‐looking, in respiratory distress with a respiratory rate of 40 breaths per minute. He had a temperature of 37.4°C with records of low‐grade pyrexia 37.6°C in his outpatient book. He was pale and had grade 1 clubbing, poor dentition, and foul‐smelling breath, and there were no other stigmata of infective endocarditis. His pulse rate was 128 beats per minute, and it was regular and full volume. He was normotensive with a BP of 114/71 mm Hg, his jugular venous pressure (JVP) was not elevated, but his precordium was active and the apex was hyperkinetic, thrusting and displaced in the 6th intercostal space anterior axillary line, there was no thrill, 1st and 2nd heart sounds were present and normal, there was a 3rd heart sound, and there was a grade 3 pansystolic murmur in the apical region radiating to the axilla. His chest was clinically clear, and he had a tender hepatomegaly and a mild splenomegaly.

**Figure 1:**
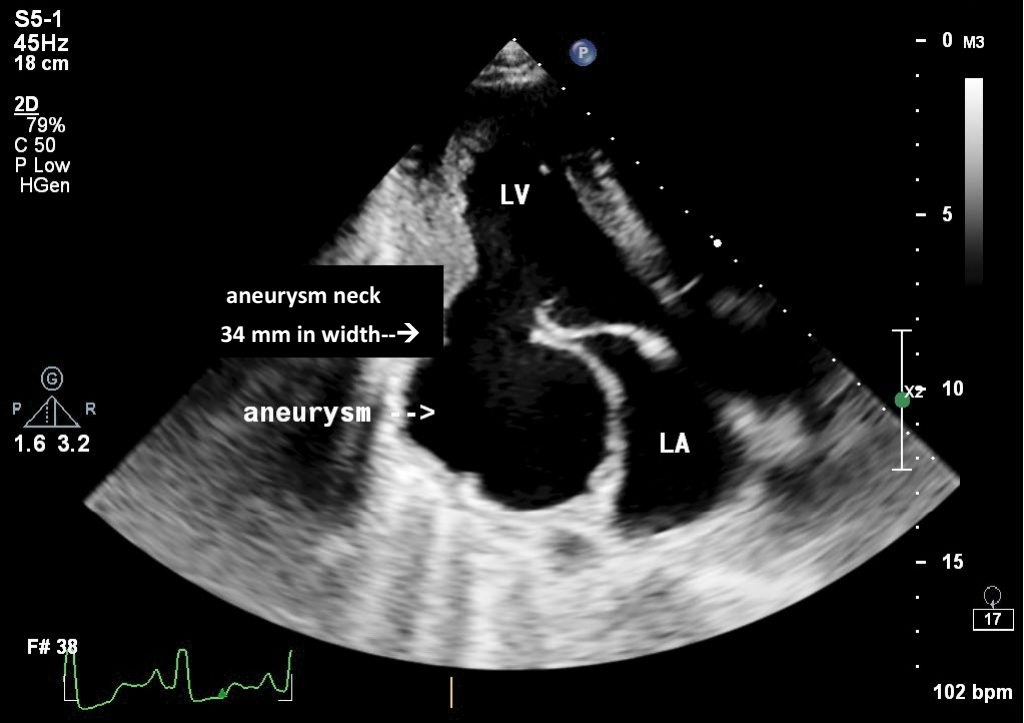
Transthoracic echocardiogram showing the large submitral valve aneurysm and aneurysm neck which is 34 mm in diameter. LA, left atrium; LV, left ventricle; RV, right ventricle

**Figure 2:**
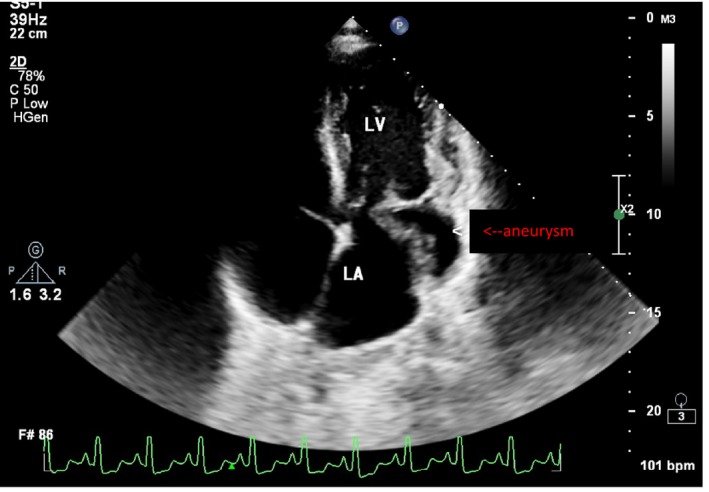
Another image showing the submitral valve aneurysm. LA, left atrium; LV, left ventricle; RV, right ventricle

**Figure 3:**
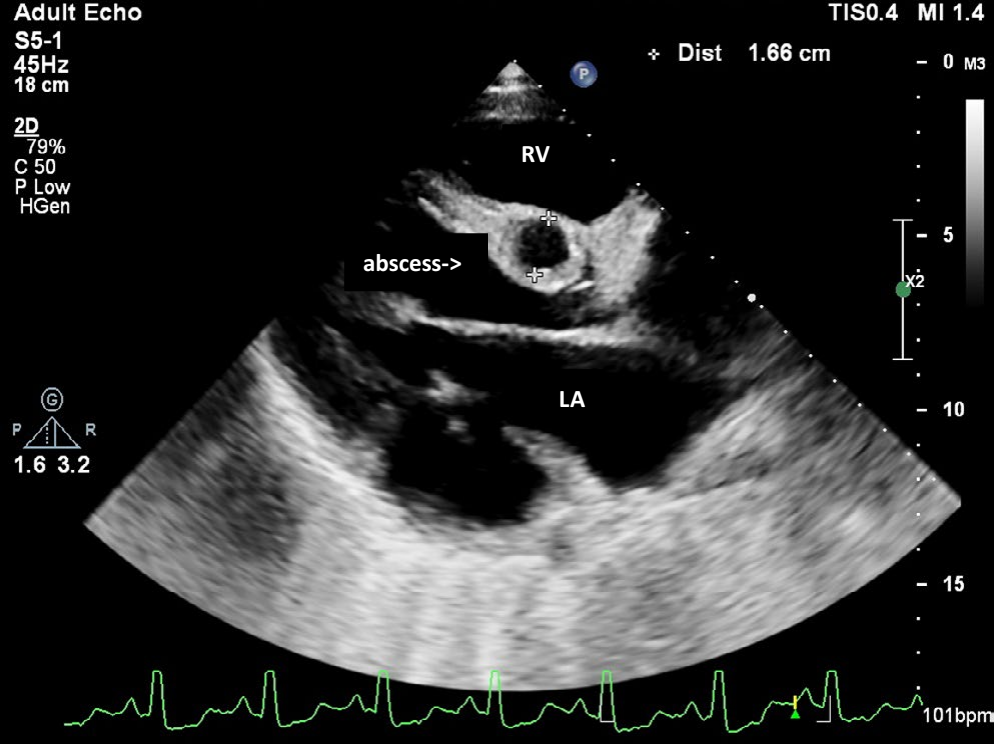
Long‐axis view of the transthoracic echocardiogram showing the interventricular septum abscess. LA, left atrium; LV, left ventricle; RV, right ventricle

We managed the patient as infective endocarditis with empirical ceftriaxone and gentamicin as well as standard antifailure treatment on the basis of the left‐sided heart failure symptoms that the patient presented with. Other differentials included tuberculosis and suppurative lung disease. The patient presented with a chest radiograph which was a week old which showed a cardiomegaly and clear lung fields. A TTE showed a large submitral aneurysm under the posterior leaflet of the mitral valve. The neck was 34 mm in diameter, and the aneurysm area was 22 mm^2^ which accommodated most of the left ventricle stroke volume (hence probably why the patient appeared to have heart failure). There was moderate mitral regurgitation due to disruption of posterior leaflet by aneurysm (see Figures 1 and 2). The left ventricle ejection fraction was 59%. There was a probable abscess in the interventricular septum (IVS) just under the aortic valve (see Figure 3). There was marked pulmonary hypertension and cor pulmonale.

He had a normal hemoglobin of 12.8 g/dL and an elevated white cell count of 18 900 mm^3^ with polymorphonuclear leukocytosis. His abdominal ultrasound scan was normal. A repeat chest radiograph showed bilateral infiltrates in both lung fields with a mottled appearance. Sputum for gene expert was negative for Mycobacterium tuberculosis. The patient was HIV negative. Blood cultures could not be obtained due to financial constraints. A discussion to move the patient to a facility where surgical options were available was made, but the patient and his relatives were unwilling due to the distance and the costs of going to a city where they did not have any relatives who could support them. We therefore continued with our treatment of intravenous antibiotics and antifailure treatment. The patient did initially show a slight improvement of symptoms; however, on day 6 of treatment, the patient developed interstitial nephritis and gentamicin had to be discontinued. He was also commenced on tranexamic acid 1 g orally once daily as he continued to have hemoptysis. The patient's condition steadily deteriorated over the next couple of days, and he subsequently developed gangrene of all his digits and on day 10 of admission succumbed to his illness on day 11.

## 3 DISCUSSION

Submitral aneurysm is a rare cardiac pathology with around a few hundred cases reported.^1^, ^3^ A study in Angola found submitral valve aneurysm in 4.1% of congenital heart disease diagnosed in patients aged greater than or equal to 15 years within 10 years.^4^

It is thought to be caused by a congenital defect in the posterior portion of the mitral annulus occurring commonly in African black patients although cases have been reported in other races globally.^5^ It can also be as result of inflammation with associations reported with Takayasu arteritis as well as infections such as HIV, tuberculosis, and infective endocarditis. Presentation is varied with patients mostly presenting at a young age. Patients may be asymptomatic but the most common presentation is moderate‐severe mitral regurgitation, resulting in gradually worsening dyspnea. Heart failure, systemic thromboembolism, ventricular wall rupture, myocardial ischemia due to compression of coronary arteries, and ventricular arrhythmias particularly ventricular tachycardia have all been reported.^1^, ^2^, ^5^

Surgical intervention is the only method of treatment of submitral valve aneurysm.^1^, ^6^ To plan for intervention, precise anatomical delineation is required and a cardiac multidetector computed tomograph (MDCT) and three‐dimensional TTE complement two‐dimensional TTE in this. Transesophageal echocardiography (TEE) is fundamental in the diagnosis of complications of submitral valve aneurysms such as rupture of the aneurysm into the left atrium, and it is recommended to perform a TEE whenever it is possible.^4^ Although it is expensive, cardiac magnetic resonance imaging is increasingly being used in the diagnosis of congenital heart diseases with advantages over other imaging modalities.^2^, ^7^

Our patient presented with symptoms of heart failure due to the submitral aneurysm occupying most of the left ventricle stroke volume and mitral regurgitation due to disruption of the posterior leaflet by the aneurysm. He also developed thromboembolic complications toward the end of his illness as he developed gangrene of all his digits. We think that in our patient the submitral valve aneurysm was as a result of subacute bacterial endocarditis. The limitations in our case were that we were unable to get a blood culture and inflammatory markers. There was also a limitation in our patient's access to surgical intervention.

## 4 CONCLUSION

Early recognition of this condition is important to prevent and treat various complications that can arise such as rupture, conduction abnormalities, aortic regurgitation, and left ventricular dysfunction.^8^

## CONFLICT OF INTEREST

None declared.

## **AUTHOR** **CONTRIBUTION**

RC: served as admitting doctor and author of the case report.
